# The hand-reversal illusion revisited

**DOI:** 10.3389/fnint.2012.00083

**Published:** 2012-09-26

**Authors:** Sang W. Hong, Linda Xu, Min-Suk Kang, Frank Tong

**Affiliations:** ^1^Department of Psychology, Florida Atlantic UniversityBoca Raton, FL, USA; ^2^Vanderbilt Center for Science Outreach, Vanderbilt UniversityNashville, TN, USA; ^3^Department of Psychology, Vanderbilt UniversityNashville, TN, USA

**Keywords:** hand-reversal illusion, visuo-tactile-motor interaction, multisensory perception, proprioception, remapping

## Abstract

The hand-reversal illusion is a visuomotor illusion that is commonly seen in children's play. When participants attempt to lift a designated finger while their hands are cross-folded, they are likely to erroneously lift the matched finger of the other hand; however, such errors are rare when subjects close their eyes. Based on the fact that the illusion disappears without visual input, researchers previously concluded that the illusion depends upon visual and proprioceptive conflict (Van Riper, 1935). Here, we re-evaluated this visual-proprioceptive conflict hypothesis by obtaining reaction time measurements because, in the original study, subjects might have relied on a strategy of responding more slowly to minimize making errors. We found that the impairment due to cross-folding one's hand persisted in the absence of the visual input, as evidenced by delayed response times (RTs). Further, we found that such impairment occurred when the fingers of only one hand were tested, indicating that the impairment was not due to left-right confusions of the hands during tactile identification or response selection. Based on these results, we suggest that the illusion is not solely due to the conflict between visual and proprioceptive information. Instead, we propose that the unusual configuration itself that involves a reversal of the left and right hands in external space also contributes to the impaired motor response.

## Introduction

One of the most important goals of sensory processing is to guide action. For example, the execution of a goal-directed movement such as grasping or pointing requires the subject to determine the location, size, and shape of the target object through sensory processing (for review, Goodale and Servos, 1996). Coordination of vision and proprioception is crucial for goal-directed hand movements (Rossetti et al., 1995; van Beers et al., 1999). Specifically, it has been shown that integration of both visual and proprioceptive information improves spatial localization performance (van Beers et al., 1999). When visual and proprioceptive information about hand position is in conflict, as can be induced by placing a wedge prism in front of the subject's eyes, the subject perceives the hand position somewhere between the vision-based and the proprioception-based location, slightly closer to the vision-based position (Pick et al., 1969; Warren, 1980; Touzalin-Chretien et al., 2010).

**Figure 1:**
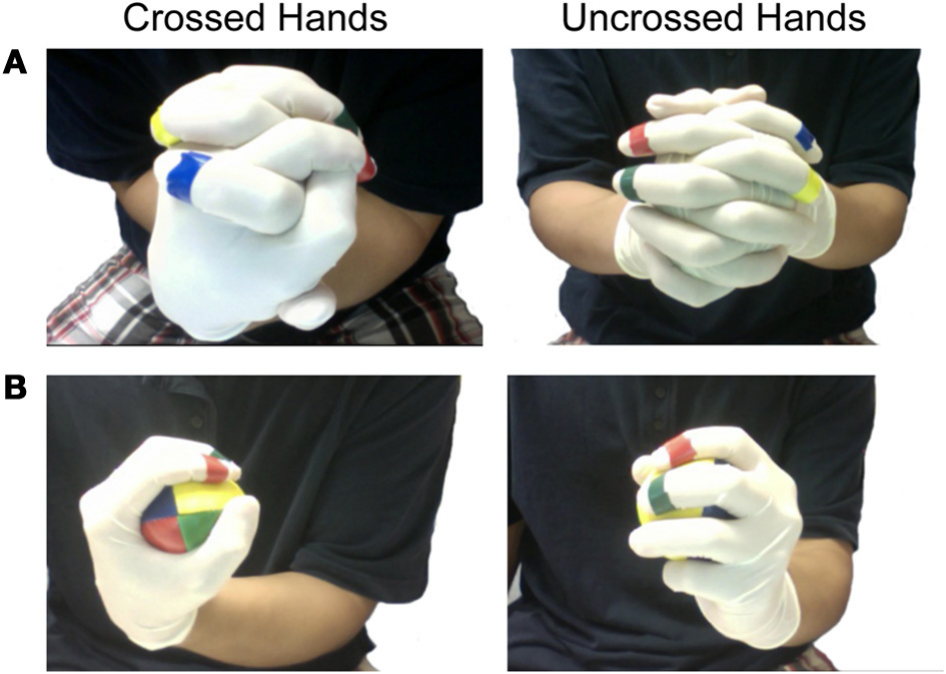
**Hand and finger postures. (A)** Example of the hand and finger posture for the Hand-Reversal Illusion (left) and a naturally folded hand posture (right). **(B)** Hand and finger postures with the ball in Experiment 3.

The hand-reversal illusion, originally called the “Japanese Illusion” (Burnett, 1904; Klein and Schilder, 1929; Van Riper, 1935) has been suggested to provide a compelling example of the importance of multisensory integration in making simple hand movements, such as lifting a finger. After folding the two hands naturally, as shown in the right panel of Figure 1A, a participant can easily lift the index finger of the left hand upon instruction. In contrast, when the two hands are cross-folded, as shown in the left panel of Figure 1A, the participant often lifts the right-hand index finger when visually instructed to lift the index finger of the left hand. This type of error in motor behavior was believed to occur because of conflict between visual and proprioceptive information: all right-hand fingers appear to belong to the left-hand and vice versa, even though one knows that the positions of the two hands have been reversed and folded based on proprioceptive information. Consistent with this hypothesized conflict between vision and proprioception, it was reported that errors were virtually eliminated if conflicting visual information was prevented by blindfolding the participant, and instruction was given solely by touching the designated finger (Burnett, 1904; Van Riper, 1935). The importance of vision in body representation can be also found in the “rubber-hand illusion” (Botvinick and Cohen, 1998; Ehrsson et al., 2004) and in mirror therapy for “phantom limb” pain (Ramachandran and Rogers-Ramachandran, 1996). For example, in clinical trials of mirror therapy, patients with traumatic amputations reported vivid kinesthetic and somatic sensations in the missing hand when looking at the mirror image of the intact hand when instructed to perform coordinated bimanual movements.

There could be, however, an alternative explanation for the hand-reversal illusion. It is possible that the hand-reversal illusion is simply due to the greater confusability of finger representations of the two hands in the cross-folded configuration, rather than the conflict between visual and proprioceptive information. It has been shown that unnatural configurations of fingers and hands, such as interweaving fingers (Zampini et al., 2005; Haggard et al., 2006; Riemer et al., 2010; Overvliet et al., 2011) and crossing hands (Benedetti, 1985; Yamamoto and Kitazawa, 2001; Heed et al., 2012), can affect tactile localization on the fingers and hands. The effect of the configuration of fingers and hands on localization and identification of touch has been observed both with (Haggard et al., 2006) and without (Riemer et al., 2010; Overvliet et al., 2011) visual information, indicating that visual information may not be the main source of confusion. Instead, these studies suggest that the effect of unnatural body configuration on localization of touch is due to the conflict between the somatotopic body coordinate and the external spatial coordinate. For example, when two hands are crossed-folded (see Figure 1A, left), the right hand belongs to the right side of the body in terms of somatotopic coordinates but is located on the left side in terms of external spatial coordinates. According to a somato-perceptual information processing model (Longo et al., 2010), the spatial location of the finger within the body schema should be first identified, which is achieved based on somatotopic organization, and then transformed to an external spatial representation to execute a finger movement. This remapping of the representation of the body part to the external spatial representation is important for performing goal-directed movements such as reaching and pointing (Sarlegna et al., 2009), since such actions require localization of both the target object and the hands in the external three-dimensional space.

In the current study, we were specifically interested in which of three factors might cause the hand-reversal illusion. One possibility, as was originally proposed by Van Riper (1935), is that potentially confusing visual information in the crossed-hands position leads to slower response times (RTs) and more errors. Another possibility is that unnatural hand configuration itself (switch between left and right body part) impairs a person's ability to localize the touched digit or to make the relevant motor action in response to that touch due to greater confusability between left and right hands. Lastly, the impairment may be due to the mismatch between the somatotopic body representation and the external spatial representation (Longo et al., 2010), as suggested by previous research on tactile localization (Haggard et al., 2006; Riemer et al., 2010; Overvliet et al., 2011). We conducted three experiments to distinguish between these possible accounts.

In our experiments, we re-evaluated the illusion by obtaining reaction time measurements because, in the original study, subjects might have relied on a strategy of responding more slowly to minimize making errors. If potentially confusing visual information is not the main cause of the illusion, we should be able to observe evidence of the illusion that is slower responses with cross-folded hands, even when conflicting visual input is eliminated. We used only tactile cues (tapping the designated finger) to directly compare the results from different visual conditions (i.e., with vs. without input). In the second experiment, we examined whether RT delays in the crossed-hands configuration was attributable to left-right confusions during response selection, by testing fingers from only one hand. Moving the finger that was touched requires localization of the tactile input, response selection of the finger to move, and execution of the movement. RT delays might occur at any of these processing stages. By testing only a single hand in Experiment 2, we minimized the potential for left-right confusion between the hands at the stage of both identification and response selection. If delays in RT are still observed in the crossed-hands position, such a result would indicate that the impairment is unlikely to reflect confusion at these stages. In the third experiment, we determined whether RT delays in the crossed-hands configuration might simply be due to the unnatural posture of the hands and fingers, by testing only one hand. Note that both hands were used for cross-folding but fingers from only one hand were tested in the second experiment. If confusability of the left and right hands is the primary cause of RT delays in the crossed-hands configuration, then no impairment should occur when only a single hand makes a similar unnatural configuration.

## Materials and methods

### Participants

Twenty healthy adult volunteers with normal or corrected-to-normal vision participated in the experiments. Each participant took part in two of the three experiments. All participants provided informed consent to participate in the study, which was approved by the Vanderbilt University Institutional Review Board.

### Procedure

For the experiment, the participant sat between two desks that each supported a MacBook Pro 13” computer used for video recording (PhotoBooth software) the participant's hands from both sides. The participant wore latex gloves, and each of the index and middle fingers were marked with a unique color band for experimental coding (Figure 1). This experiment focused on the index and middle fingers exclusively, as it proved more difficult to move the third or fourth digits independently, especially when the hands were positioned in the reversed configuration.

An experimenter stood in front of the participant and waited for the instruction generated by a separate MacBook Pro 15” computer, which indicated the specific finger to be tested on each trial. When the experiment began, an assistant started video recording the participant's arms, hands, and fingers; the camera viewpoint was adjusted so that other body parts remained out of view, including the face. The recording frequency was set to 30 frames per second. The assistant also started a computer program that showed the experimenter which finger to tap on each trial, by presenting pictures of the hands and the designated colors. An auditory beep occurred 3 s after the picture was displayed to the experimenter, cuing the experimenter to use a hard plastic pen tip to touch the relevant finger between the first joint from the fingertip and the second joint. The beep also served as a temporal cue to prepare the participant, and was presented in every experimental condition. The participant's task was to lift the tapped finger as quickly as possible without making errors.

RT for lifting a finger on a given trial was measured by counting video frames. Each frame was calculated as 33 ms with a 30 frame/s recording frequency. The starting point for counting frames was defined as time when the pen tip first touched the finger, and the end point was defined as the first frame that showed a finger rising away from the back of the folded hands. The frame count included both the starting and end points. RT was calculated by multiplying the frame count by 33 ms. Since overall RTs differed considerably across participants, RTs were normalized by each individual's mean reaction time in the experiment, resulting in a value greater than 1 for slower responses and a value lower than 1 for faster responses.

The experiment consisted of a 2 × 2 design, with the participant's hands arranged in a normal or reversed configuration and the eyes open or closed. In the eyes-open condition, participants were asked to look steadily at their hands; in the eyes-closed condition, participants were instructed to keep their eyes closed. In the uncrossed hands condition (Figure 1A, right), participants folded their two hands naturally. In the reversed hands configuration (Figure 1A, left), they crossed the wrists, interlaced the fingers with the thumbs pointing downwards, and then turned the arms and hands in and around toward the body until the fifth fingers were closest to the body and the thumbs pointed upwards and outwards. The RT for lifting the fingers was measured eight times for each of the four fingers and the order of the 32 finger taps in each condition was completely randomized. It took about 5 min to complete one condition.

In Experiment 1, we tested the original hand-reversal illusion with two hand positions: cross-folded hands (Figure 1A, left), and uncrossed hands (Figure 1A, right). In Experiment 2, participants were instructed to make the same hand positions as in Experiment 1, but the fingers of only one hand were tested to minimize potential left-right confusion between the hands. In Experiment 3, participants grasped a ball with their dominant hand. In this position, participants mimicked the cross-folded and uncrossed hand positions (Figure 1B). By using only one hand, we could further remove left-right confusion, so that we were able to test whether simple conflict between the somatotopic representation of a single hand and its position in external space would be sufficient to induce the impairment. The two hand positions (cross-folded and uncrossed) were tested in combination with the two visual conditions (eyes open and closed) for all three experiments, resulting in a total of 12 different conditions. Each subject performed two (out of three) randomly chosen experiments. Within each experimental session, the order of four conditions was randomized.

## Results

**Figure 2:**
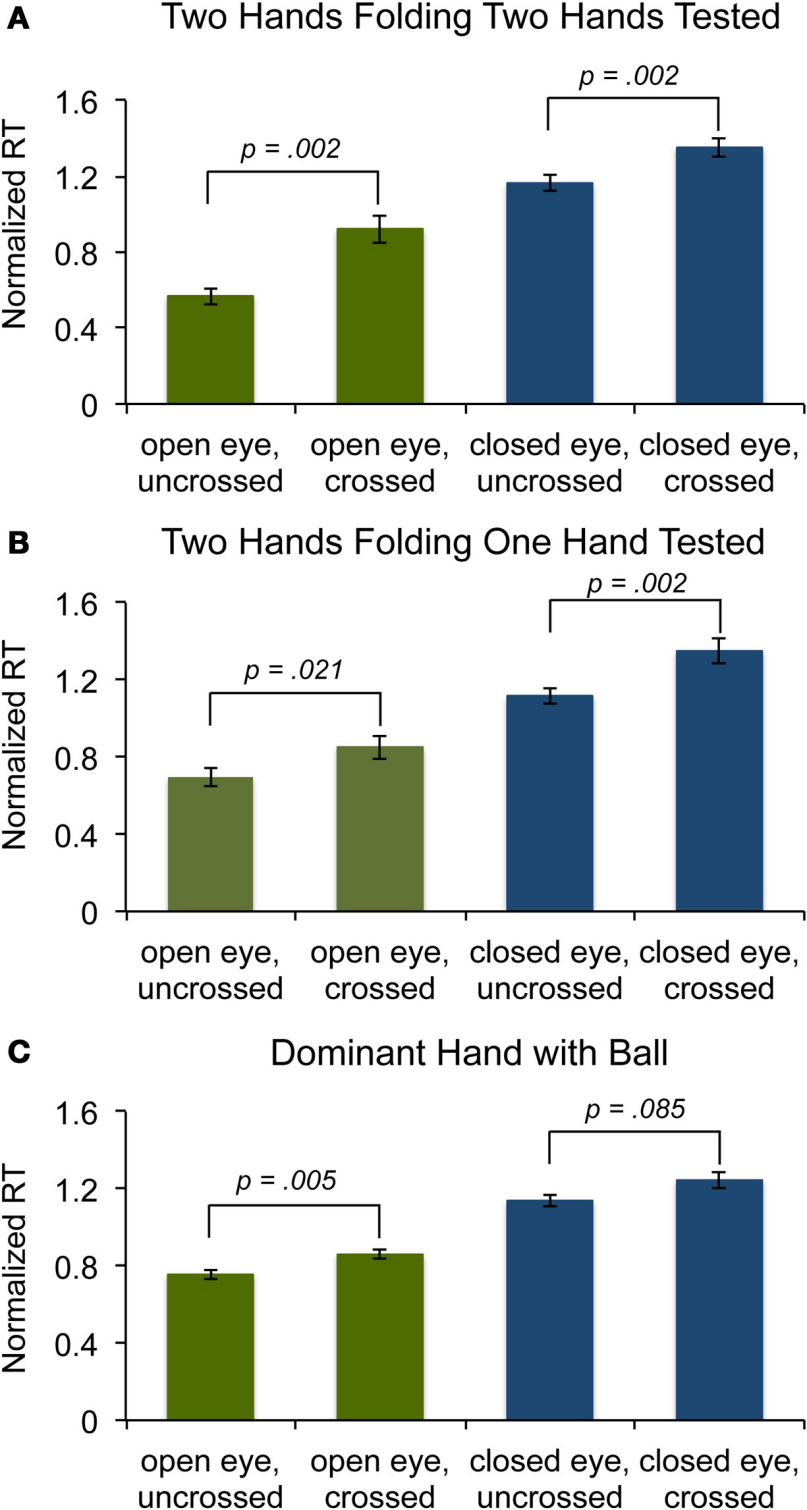
**Results from Experiments. (A)** Experiment 1: Normalized reaction time (RT) for Hand-Reversal Illusion with open (green) and closed eyes (blue). Error bars represent the standard error across participants. **(B)** Experiment 2: Same as **(A)** but tested fingers from only one hand. **(C)** Experiment 3: Same as **(A)** but with only one hand grabbing the ball.

We measured how long it took participants to move the relevant finger that was tapped under the following conditions, with eyes opened or closed and with hands folded in a normal or reversed configuration. Normalized RTs are shown in Figure 2A. The RT was measured 32 times for each condition, and only correct responses (lifting the finger indicated by the experimenter) were used for analysis. Incorrect responses were very rare for all participants (one or two errors, if any, for the cross-folded hand condition). The very low frequency of errors was due to the fact that the relevant finger was directly touched in these experiments.

A Two-Way analysis of variance (ANOVA) within-subject design revealed a significant main effect of both hand position (cross-folded and uncrossed) [*F*_(1, 9)_ = 34.24, *p* < 0.01] and eye condition (open and closed) [*F*_(1, 9)_ = 34.74, *p* < 0.01]. The faster RTs in the open-eye condition might reflect an overall advantage of multi-sensory information on localization performance (Kennett et al., 2001; Forster et al., 2002). However, we did not find evidence of a significant interaction between two conditions [*F*_(1, 9)_ = 3.41, *p* = 0.10], indicating that the hand-reversal illusion occurred regardless of whether the eyes were open or closed and that the conflict visual information did not provide additional source of confusion when tactile cue was used. A planned orthogonal contrast confirmed that RTs were significantly slower in the hands-crossed configuration than in the normal configuration, both when participants had their eyes open [*F*_(1, 9)_ = 19.22, *p* < 0.01] and when their eyes were closed [*F*_(1, 9)_ = 18.61, *p* < 0.01].

The visual conflict hypothesis cannot explain these results, as we find that the hand-reversal illusion, in terms of RT, persists without visual input. Instead, this result indicates that the unnatural hand posture itself causes difficulty in localizing or responding with the designated finger to lift. This proved true even though the designated finger was directly touched, thereby providing unambiguous information about the relevant finger to move. Previous studies on tactile localization with unnatural hand configuration have found that mismatches between the body schema representation for the left and right hands, and their location in external space (left and right side from the body center) may cause difficulty in one's ability to localize a tactile stimulus.

Lifting an indicated finger involves multiple stages of processing, including identification of tactile input, response selection and execution of finger movement. RT delays could occur at any or all processing stages. To examine whether the confusion occurs at the stage of tactile identification or response selection or execution of movement, we measured RTs to test probes presented exclusively to the fingers of just one hand. With this experiment, potential left-right hand confusion at the level of tactile identification and response selection can be minimized, since participants have to use fingers from only one hand. A Two-Way within-subject ANOVA revealed that the main effect of the hand position was significant [*F*_(1, 9)_ = 17.84, *p* < 0.01], indicating that slower RTs in crossed-hands configuration was unlikely to reflect confusions at the processing stage of identification or response selection (Figure 2B). A planned orthogonal contrast confirmed that RTs were significantly slower in the hands-crossed configuration than in the normal configuration for both eye conditions (open: [*F*_(1, 9)_ = 7.81, *p* < 0.05] and closed: [*F*_(1, 9)_ = 19.27, *p* < 0.01]).

In the crossed hands configuration with participants' own two hands, the impairment in response latency could occur due to the confusion in the bodily representation of handedness (left-right hands). Alternatively, it remains a possibility that simple conflict between the somatotopic representation of a single hand and its position in external space would be sufficient to induce the impairment without confusion in left-right hands. To address this potential concern, we conducted a control experiment that required participants to use only a single hand. We hypothesized that the use of a single hand should further minimize the left-right confusions when participants attempted to plan the correct motor action, but conflict between somatotopic representation of the hand and its position in external space remains. Participants were instructed to make a hand posture similar to that required for Experiment 1, by holding a ball in the palm of their dominant hand (Figure 1B). We tested participants' dominant hand since we found no difference in reaction times between the two hands for the participants in Experiment 1. A Two-Way within-subject ANOVA revealed a significant main effect of both eye condition [*F*_(1, 9)_ = 73.16, *p* < 0.001] and hand position [*F*_(1, 9)_ = 17.81, *p* < 0.01], indicating that the impairment in finger responses still occurred when only one hand was positioned in a reversed configuration over the body midline (Figure 2C). A planned contrast showed that, however, RT was significantly slower for crossed hand condition with open eye [*F*_(1, 9)_ = 13.48, *p* < 0.01]. In closed eye condition, RT was slow overall for crossed hand but was not significant [*F*_(1, 9)_ = 3.73, *p* = 0.085].

## Discussion

In this study, we investigated possible explanations for the hand-reversal illusion, also known as Japanese Illusion, other than visuo-proprioceptive conflict hypothesis. When RTs, instead of error rate, were measured, we found that the impairment in finger response persisted even after conflicting visual information was eliminated. This result indicates that, contrary to the long-standing belief, the conflict between visual and proprioceptive information is not the only cause of the illusion. Instead, we propose that the hand-reversal illusion can be understood within the same framework that explains the effect of various unnatural hand configurations on tactile perception.

Previous research has shown that temporal order judgments (TOJ) are less precise for tactile stimulation delivered to the two hands when those hands are crossed and positioned in the contralateral hemifield, compared to when the two hands are normally positioned (Yamamoto and Kitazawa, 2001; Shore et al., 2002; Schicke and Röder, 2006). This hand-crossing effect has been interpreted as evidence of a conflict between the somatotopic body representation (e.g., right hand) and the representation of that body part in external space (e.g., left side of one's body), which can result in a difficulty in tactile processing. Resolution of this conflict may be required to execute a correct action (Heed et al., 2012). Also, interweaving one's fingers can disrupt the precise localization of which finger was touched, the spatial sequence of multiple touches (Haggard et al., 2006; Riemer et al., 2010; Overvliet et al., 2011) and the discrimination of tactile stimulation (Zampini et al., 2005). These previous studies are generally consistent with the present findings.

From this previous work, however, it was not clear whether a reconciliation between somatotopic representation and external spatial representation would be necessary for making a simple finger movement in response to local tactile stimulation. The present study required participants to respond directly to local touch with a finger movement. Our experimental procedure therefore avoided requiring participants from having to make an explicit judgment regarding the location of the cued body part according to external spatial coordinates. Nevertheless, we observed a behavioral cost in RTs for the crossed-hands position.

Previous studies, however, had not tested whether such conflict would occur when only one hand is located in the contralateral side from the body center. If the mismatch between somatotopic body representation and the external spatial representation is the real cause of the crossing effect, the effect should persist when only one hand crosses over the body midline in external space. Our result support this hypothesis by showing that RTs to the tactile cue are slower even when only one hand was tested, indicating that simple conflict between the somatotopic representation of a single hand and its position in external space is sufficient to induce the impairment.

In the current study, we were able to rule out the processing stages of tactile identification and response selection as a possible locus for the confusion, but it remains to be determined whether the impairment occurs at the stage of motor planning and execution (sending the motor command to move the finger). Of potential relevance, Shore et al. (2002) found that when a cue was provided in the visual domain (i.e., an LED light on the designated finger) instead of the tactile domain (i.e., a tap on the designated finger), the effect of hand-crossing on TOJ was reduced. This result suggests that the left-right confusion occurred at the tactile localization stage rather than at the execution stage. In contrast, the hand-reversal illusion has been found to be stronger when instruction is given through visual rather than tactile cues (Burnett, 1904; Van Riper, 1935). This discrepancy may suggest that the confusion occurs at the stage of motor planning in the hand-reversal illusion. Consistently, we observed delays in RT when any possibility of confusion in the stage of identification and response selection was minimized by testing fingers from only one hand (Experiment 2). The result suggests that behavioral cost in finger movement with cross-folded hands may occur at the stage of motor planning and execution.

The hand-reversal illusion has been believed to occur due to visual-proprioceptive conflict since errors in finger lifting response are virtually abolished without visual input. Here we show that the visual-proprioceptive conflict may not be the only cause of the illusion. Although, it has been reported earlier and replicated here again that errors in lifting indicated fingers almost never occur with tactual cue, our results suggest that the cost of unnatural hand configurations, shown by delays in RT, persists. Unnatural hand configurations can induce impairment in localization of finger with visual cue (Shore et al., 2002) as well as tactile cue. Together with our results, we suggest that the unnatural hand configuration itself (reversal of between left and right body parts), which induces conflict between the somatotopic body representation and representation of the body in the external space, also contributes to the hand-reversal illusion.

### Conflict of interest statement

The authors declare that the research was conducted in the absence of any commercial or financial relationships that could be construed as a potential conflict of interest.
